# The role of Raman spectroscopy in biopharmaceuticals from development to manufacturing

**DOI:** 10.1007/s00216-021-03727-4

**Published:** 2021-10-20

**Authors:** Karen A. Esmonde-White, Maryann Cuellar, Ian R. Lewis

**Affiliations:** Endress+Hauser, 371 Parkland Plaza, MI 48103 Ann Arbor, USA

**Keywords:** Raman spectroscopy, Cell culture, Protein higher-order structure, Upstream bioprocessing, Downstream bioprocessing, Biopharmaceutical

## Abstract

Biopharmaceuticals have revolutionized the field of medicine in the types of active ingredient molecules and treatable indications. Adoption of Quality by Design and Process Analytical Technology (PAT) frameworks has helped the biopharmaceutical field to realize consistent product quality, process intensification, and real-time control. As part of the PAT strategy, Raman spectroscopy offers many benefits and is used successfully in bioprocessing from single-cell analysis to cGMP process control. Since first introduced in 2011 for industrial bioprocessing applications, Raman has become a first-choice PAT for monitoring and controlling upstream bioprocesses because it facilitates advanced process control and enables consistent process quality. This paper will discuss new frontiers in extending these successes in upstream from scale-down to commercial manufacturing. New reports concerning the use of Raman spectroscopy in the basic science of single cells and downstream process monitoring illustrate industrial recognition of Raman’s value throughout a biopharmaceutical product’s lifecycle. Finally, we draw upon a nearly 90-year history in biological Raman spectroscopy to provide the basis for laboratory and in-line measurements of protein quality, including higher-order structure and composition modifications, to support formulation development.

**Graphical abstract:**

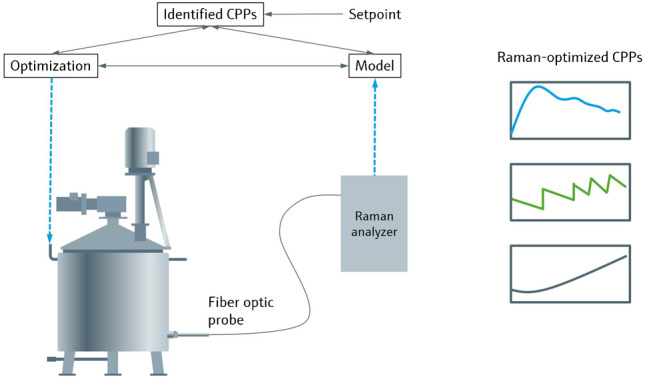

## Introduction

### What is bioprocessing

Biotechnology was originally a term for agriculture that was introduced by agricultural engineer Karl Ereky in 1919 [1]. Biotechnology describes a biological transformation process to make raw materials into something more useful, where the biological transformation can be used to create a new product or to eliminate undesirable aspects of a material. Creating a new product through biological transformation is not new to humans, as the earliest use of biotechnology was fermentation to preserve food or beverages [2]. Extension of biotechnology into various industries grew in the late 1900s as knowledge in molecular and cell biology, culture technology, and analysis grew. As early as 1982, it was recognized that industrial biotechnology processes to produce polymers or chemicals can reduce reliance on fossil fuel processing and offer the potential to balance productivity with costs [3]. Today, biotechnology principles are used to create products including everything from polymers, fermented foods, fuels, and medicines as well as bioremediation of heavy metals or colorants in wastewater treatment [4, 5].

Extension of biotechnology principles to cell-based production of therapeutic macromolecules has revolutionized the field of medicine in the types of active ingredient molecules and treatable indications. These therapies, known as biopharmaceuticals, are highly specific and highly efficacious. They are not possible to produce using traditional synthetic chemistry approaches because of their size and complex higher-order structure. Instead, a host cell is genetically engineered to express the target macromolecule. While monoclonal antibodies are the dominant type of biopharmaceutical molecule, biopharmaceuticals can encompass many molecule types including antibody–drug conjugates, bispecific antibodies, amino acids, vitamins, nucleic acids, enzymes, vaccines, and cell-based products. Biopharmaceuticals are used for various indications such as cancer, autoimmune disorders, cardiovascular conditions, inflammatory diseases, and diabetes [6]. Today, there are over 300 approved biopharmaceutical products on the market, consisting mostly of monoclonal antibodies (mAb) produced by mammalian cells [6]. Biopharmaceuticals need to be expressed by the host cell, isolated, purified, and then formulated to ensure a consistently safe and efficacious medicine. Bioprocess engineering or bioprocessing describes how the principles of biotechnology get translated into creating useful objects [7]. Bioprocessing is the phrase most commonly used to describe how biopharmaceutical products are created. Briefly, upstream bioprocessing describes a series of cultivations to grow cells, typically in suspension, in culture conditions to a set amount of total and viable cells. Downstream bioprocessing is a series of processing steps to isolate and purify the molecular target from the cell.

### Raman spectroscopy as a process analytical technology (PAT) in bioprocessing

Advances in cell engineering, process control, and media composition are credited with improving the volumetric yield of cell culture bioprocesses, making biopharmaceutical manufacturing more cost-effective and practical [8]. Adoption of PAT and Quality by Design (QbD) principles is an important contributor to improvements in bioprocess control. PAT provides real-time understanding which helps to manage risk throughout a biopharmaceutical product’s lifecycle. The PAT framework is an integrated approach using historical process knowledge, modeling, and analyses. Many types of physical and chemical analyses are used for bioprocessing. Traditional parameters such as pH, temperature, dissolved oxygen, feed composition, and feed timing are measured in situ. Biochemical parameters such as nutrients, metabolites, amino acids, proteins, cell viability, and biomass can be measured by spectroscopy, electrochemical sensors, biochemical assay, or chromatography. These biochemical PATs can be used in situ, integrated with an automated sampler for at-line measurements, or off-line. Spectroscopy PAT techniques are based on light’s interactions with materials. They provide a fast, label-free, non-invasive, and non-destructive chemical analysis of a material. Focused beam reflectance measurement (FBMR), ultraviolet/visible (UV/Vis), near-infrared (NIR), infrared (IR), and Raman are widely used in the pharmaceutical industry and are now mature technologies for small molecule pharmaceuticals. Vibrational spectroscopy analyses, NIR, IR, and Raman, are powerful tools in understanding composition and molecular structure. NIR uses near-infrared radiation, IR uses infrared radiation, and Raman uses visible or near-infrared radiation to measure chemical composition and molecular structure. Vibrational spectroscopy has been applied to industrial bioprocessing since the late 2000s, with Raman spectroscopy first described in industrial upstream applications in 2011 [9].

Raman spectroscopy is based on optical radiation interacting with molecular vibrations resulting in the exciting radiation becoming inelastically scattered. Molecular vibrations refer to the motion of a molecule in a way that the center of gravity does not change. Molecular vibrations take on several possible variations including bending, stretching, rocking, wagging, twisting, or scissoring. It is important to know that different parts of a larger molecule, such as proteins, can have different vibrations. Optical photons can interact with molecular vibrations. When scattering occurs, there are three possible outcomes: the first outcome results in the creation of scattered photons having the same energy as the incoming photon; the second outcome results in the scattered photons losing some energy (resulting in a red-shifted wavelength); and the third outcome results in the scattered photons gaining energy (resulting in a blue-shifted wavelength) [10]. Elastically scattered means that the radiation is scattered without changing its color. Inelastically scattered light, where the radiation is scattered and the wavelength is red-shifted because it loses a little bit of energy after interacting with a vibrating molecule, is termed Stokes scatter. In rare cases, the radiation will scatter and gain a little bit of energy, as is the case for anti-Stokes Raman spectroscopy [11, 12]. The fundamental process that produces a Raman signal offers advantages such as high specificity, as well as disadvantages such as the feebleness of the phenomenon and competition with fluorescence.

The “excessive feebleness of the effect” was acknowledged in the original paper by Raman and Krishnan [10]. And there have been efforts since then to enhance Raman signal and reduce the effect of fluorescence. Enhancement approaches are described later in the “Raman variants” section of this paper. Approaches to reduce fluorescence can be achieved through hardware modification and, with the advent of computerized spectral collection, through software [13]. Hardware modifications approaches include localizing the Raman sampling volume through surface-enhanced Raman or tip-enhanced Raman, shift the excitation wavelength to longer wavelengths, or modulate the fluorescence either through frequency modulation or time-resolving the signal. Software-based approaches include background correction algorithms or derivative spectroscopy. A hybrid hardware and software approach is shifted-excitation Raman difference spectroscopy (SERDS), where the sample is sequentially measured by two lasers close in wavelength and then the slight differences are subtracted in software.

Despite its drawbacks, Raman spectroscopy is a technology with industrial value in laboratory or process environments across many industries. The specificity provided by Raman is akin to a fingerprint, which is a very powerful tool for identification, quantification, and change monitoring uses. Because this Raman-based “molecular fingerprint” can often be obtained without sample preparation in aqueous environments, the technology is amenable for direct, real-time, in-line bioprocess measurements.

Raman spectroscopy has inherent advantages over NIR and IR in bioprocessing applications because of its specificity, compatibility with aqueous systems, and sampling flexibility. Since its introduction to industrial settings in the mid-1990s, Raman spectroscopy based on modern dispersive instrumentation has been used to solve identification, quantification, and process monitoring problems. The first applications were closely linked to those already analyzed by Fourier-transform infrared spectroscopy (FTIR) or FT-Raman since Raman provides compositional and molecular structure information similar to FTIR. It was typical to see in those early reports that Raman was tested only after the implementation limitations of FTIR or FT-Raman, including sample probe fouling, high laser power, or incompatibility with aqueous systems, were recognized. With those first successes, industrial confidence in the Raman technique and the robustness of the hardware grew, and more applications were reported for Raman-based product or process understanding. Since then, Raman has proven to provide the specificity of FTIR with the measurement ease of near-infrared (NIR) spectroscopy. A more modern perspective on Raman is that it is a first-choice PAT, rather than a PAT of last resort.

## Industrial perspectives

### Regulatory framework

Two trends in the regulatory landscape have created new scientific opportunities and financial motivations for using process analytical technologies (PAT) in small molecule and biopharmaceutical manufacturing. The first trend is increased inspection and enforcement, post-approval inspections, records inspection, and compliance with requiring generic drug manufacturers to register with the FDA. In 2015, the FDA reported a 60% increase in preapproval inspections of generic drug manufacturers between 2011 and 2013 [14]. The second trend is continued emphasis toward using Quality by Design (QbD) principles in manufacturing. In 2002, the US Food and Drug Administration (FDA) launched an initiative to encourage innovation in manufacturing technology and quality system approaches. This resulted in the 2004 FDA PAT framework and the European Medicines Agency (EMA) guidance documents on process PAT, QbD, and real-time release testing. International Conference on Harmonization (ICH) Q8, Q9, Q10, and Q11 documents have been implemented in the USA, European Union, and Japan since 2009. Briefly, Q8 (R2) and Q11 outline how to develop a control strategy to support product development, launch, and registration, Q9 discusses the application of risk-based principles, and Q10 discusses an effective pharmaceutical quality system and the commercial phase of the product. Together, these documents provide guidance on manufacturing approaches for the pre-market and commercial phases of pharmaceutical product development.

There is continued guidance on risk-based manufacturing to facilitate industry-wide adoption of PAT and QbD principles throughout a product lifecycle. ICH Q12 was released in late 2019 and provides guidance for post-approval changes. The goals of Q12 are to reduce regulatory burden and better manage post-approval changes to Chemistry, Manufacturing, and Controls (CMC) into a Post-Approval Change Management Protocol (PACMP) and Product Lifecycle Management so that manufacturers can realize flexibility and continuous improvement. Categorization of Established Conditions, input/outputs that need to be controlled to ensure product quality, can be based on input parameters such as material attributes or on output performance of models or PAT. While there is some overlap in Q12 with design space and control strategy aspects of Q8, the Q12 document specifically addresses post-market changes. More recently, ongoing work on ICH Q14 provides continuity to the ICH Q2[1] document on “Validation of Analytical Procedures: Text and Methodology.” The revision will address a multivariate analysis of spectroscopy or spectrometry data, and newer topics on analytical development such as real-time release testing. This evolving framework opens opportunities to adapt new technologies and new process understanding with potential benefits of reduced regulatory burden and improved plant availability.

### Industry-wide collaboration

Industry working groups and professional societies provide avenues for collaboration to address industry-wide challenges and opportunities. These organizations establish best practices, hold educational workshops and conferences, recognize excellence, create partnerships, provide technology roadmaps, coalesce subject matter expertise, and give practical guidance. They also publish books, release guides to help achieve compliance, and provide consensus statements on technological aspects of manufacturing. There are many regional, national, and international professional societies, working groups, and industry associations. Two groups bear mention because of their strong support for PAT and QbD within the bioprocessing industry. The Good Automated Manufacturing Practice (GAMP) subgroup of the International Society of Pharmaceutical Engineers (ISPE) routinely releases good practice guides on many topics such as computerization, calibration management, data integrity, process control systems, and management records. BioPhorum is an industry group that is committed to industry-wide sharing. Their recent papers on real-time release and continuous manufacturing approaches underscore the importance of PAT in achieving robust bioprocessing, offer practical guidance on user requirement specifications, and provide a gap analysis for a generic bioprocess producing mAb [15, 16]. Notably, they recognize the importance of in-line monitoring for supporting batch and continuous manufacturing approaches, and affirm that continuous manufacturing is only possible with in-line monitoring. In the USA, the National Institute for Innovation in Manufacturing Biopharmaceuticals (NIIMBL) supports collaboration between pharmaceutical companies and government agencies such as the FDA and National Institutes of Standards and Technology (NIST). Their 10-year roadmap seeks to address manufacturing cost, robustness, improved control, and supply flexibility [17]. These forums strongly support collaboration, embrace the PAT/QbD framework, and provide practical insight through their guides and papers.

### Initial feasibility assessment: factors to consider

Today, the answer to the question of whether a measurement problem may be well suited to Raman spectroscopy is generally “yes.” Hardware reliability, a myriad of sample measurement modalities, model transferability, and low operational costs make Raman spectroscopy an attractive analysis tool. There are scientific and environmental aspects of application feasibility. Scientific aspects of an application to consider include measurable effect size, sample concentration, optical scattering, molecular structure, and limits of detection. In some cases, there may be established user requirement specifications to assess measurement feasibility and performance [15]. For process applications, there are additional factors such as return on investment, sampling requirements, cycle time, the information encoded into the spectrum, instrument reliability and calibration, serviceability in the field, and integration into automation platforms [12]. A fast and easy way to assess scientific aspects of application feasibility is to compare it with similar well-documented applications. In this respect, scientific feasibility assessment in the life sciences benefits from a rich literature history since the 1930s. Today’s modern equipment means that many reported applications can be repeated successfully, even those first reports on structural analysis of biological molecules. Well-documented literature for life science includes applications from single-cell analysis or small molecule crystallization screening in the laboratory to cross-scale process control in active pharmaceutical ingredient (API) and biopharmaceutical manufacturing.

## Overview of instrumentation and data analysis

Raman instrumentation continues to become more amenable for non-specialist use, facilitating its use in manufacturing environments and enabling new discoveries in laboratories. Since our critical review of Raman spectroscopy in pharmaceutical manufacturing and bioprocessing in 2016 [18], we have seen developments in the use of Raman variants or enhancement strategies in basic science applications, expansion of in situ Raman measurements in upstream bioprocessing, and increasing compatibility of process Raman with process automation.

### Raman variants

When the phrase Raman spectroscopy is used, it typically refers to unenhanced spontaneous Raman spectroscopy measured using dispersive instrumentation coupled with a microscope, handheld or portable unit, or fiber-optic measurement probes. Since 2016, there have been more reports of Raman variants in the literature on applications relevant to bioprocessing, so it is worthwhile to provide an overview of relevant variants of Raman. The major classes of variations in Raman hardware are fluorescence reduction approaches, enhancement approaches, non-linear Raman, and chiral or enantioselective Raman.

Fluorescence reduction approaches for Raman spectroscopy using hardware include using red or deep-red laser wavelengths, time-resolved Raman, or shifted-excitation Raman difference spectroscopy (SERDS). The use of red or deep-red lasers is a straight-forward approach and provides suitable fluorescence reduction for many industrial applications. Yet, using a longer wavelength does require longer signal integration times because the Raman effect is inversely proportional to the excitation wavelength to the 4^th^ power. Time-resolved or SERDS may provide a faster means of reducing fluorescence, and these approaches show promise in reducing fluorescence in Raman spectra of biological materials [19, 20].

SERDS involves using two separate lasers with slightly different wavelengths to collect two distinct Raman spectra, and then those spectra are subtracted. The resulting subtracted spectra should have sufficiently reduced fluorescence. This approach is widely used in handheld Raman spectroscopy for materials identification, remote explosives or chemicals identification, or mineral studies [21]. Extension of the technique to biological applications is complicated by non-linear changes in fluorescence with wavelength. A recent paper by Cordero et al. [22] shows the use of SERDS for biological material measurements, and their results underscore practical challenges of implementing SERDS for in vitro and in vivo clinical applications. They concluded that use of a software algorithm, extended multiplicative scatter correction (EMSC), enabled better fluorescence reduction without needing to use complicated instrumentation. Additionally, the EMSC-based correction provided better signal/noise because a single, longer, measurement can be collected in the same time to collect a single SERDS measurement. Additional work has shown that taking the derivative of the Raman spectrum effectively removes broad spectral features while retaining sharp features [22].

Time-resolved Raman, more recently called time-gated Raman, adopts principles from ultrafast spectroscopy. Time-resolved Raman operates on the principle that optical scattering occurs on the picosecond time frame while fluorescence occurs on a longer, nanosecond, time frame. Rapid “gating” of fluorescence is achieved either through pulsed lasers, electronic timing of the detector, and/or filtering. Representative examples of a Kerr gate approach by Matousek et al. [23] pulsed laser and intensified charge-coupled device (iCCD) detector gating approach by Ariese et al. [24] and then gating using a single-photon avalanche detector (SPAD) [25] show the variety of approaches possible. The SPAD approach has the advantage over iCCD or Kerr gate by means of simplified optics, a smaller footprint, and higher detector efficiency. Like all approaches, time-gated Raman and SERDS are not without their disadvantages. These approaches are at an early technology development stage, appear to offer no compelling advantage over spontaneous Raman at higher wavelengths or software-based algorithms for fluorescence suppression, and appear to be currently limited to off-line or laboratory use [22, 26].

Enhancement approaches for Raman spectroscopy can increase the Raman signal, reduce the limit of detection, and reduce the sampled volume. Enhanced variants of Raman include resonance Raman, surface-enhanced Raman (SERS), surface-enhanced resonance Raman (SERRS), and tip-enhanced Raman (TERS). Resonance Raman, or UV-resonance Raman, utilizes laser wavelengths typically in the UV region to not only increase the Raman efficiency, but also overlap selected vibration state with the electronic transition state. Deep UV Raman uses UV wavelengths for excitation, but to achieve the measurement without resonance then the sample cannot have chromophores with an electronic transition in the UV. As such, non-resonant deep UV Raman is used primarily in Earth and Martian mineralogy.27, 28. There is a resonance effect when using deep UV excitation wavelengths for measuring biological materials, such as those found in a cell culture or fermentation. The combined resonance and Raman efficiency effects with lower wavelength excitation can increase the intensity of Raman bands up to 10^6^.[29–31] Resonance Raman has been harnessed to understand biological pigments, such as carotenoids, and protein higher-order structure or dynamics without the need to use exogeneous labels. SERS and TERS employ nanosized 2D or 3D metallic structures which generate a localized surface plasmon upon laser excitation. If a Raman-scattering molecule is in close contact with that surface plasmon, the Raman signal is enhanced from an electromagnetic interaction. Recent reviews provide an introduction to the measurement principles, highlight advances in metallic substrates, and discuss application areas of SERS and TERS [32, 33]. Both SERS and TERS are valuable for nanoscale analysis with applications in cell-ligand binding, single-cell dynamics, hyper-localized molecular structure, or intracellular studies. An acknowledged limitation of SERS is translating the powerful enhancement into a reliable and quantifiable measurement. A recent mini-review by Bell et al. [34] provided key parameters and recommendations, such as the use of an internal standard, to improve intra-laboratory comparison of SERS results. We anticipate that continued emphasis on reliability and quantification, and promising research into metallic nanoparticles and nanostructures, will contribute to new industrial uses of SERS in the near future [32].

Non-linear Raman and chiral Raman have powerful but niche uses. There are a number of non-linear variants of Raman spectroscopy but only some of these have been applied to biopharmaceutical applications; chief among them are coherent anti-Stokes Raman (CARS) and stimulated Raman scattering (SRS). Biological applications for CARS and SRS are rapid, non-invasive, and label-free microscopy or imaging of cells or tissues to measure lipid content. Readers are referred to several resources for more information on the theory, instrumentation, and applications of these non-linear variants [35–37]. Raman optical activity, also called vibrational circular dichroism or chiral Raman, uses circularly polarized light to measure stereochemistry from the vibrational spectrum. Raman optical activity is used to understand chirality, with applications in biomolecules including mAbs [38, 39]. Carrying out SERS, TERS, resonance Raman, Raman optical activity, and non-linear Raman experiments requires specialized equipment and we find that these variants are more commonly used in the laboratory for basic science research, with limited-to-no reported industrial process monitoring applications.

### Instrumentation for Raman spectroscopy

With respect to dispersive instrumentation, the basic components of a spectrometer are an excitation laser, optics or fibers to bring the excitation laser light to a sample, optics or fibers to collect Raman-scattered photons, and a spectrograph. The spectrograph’s functions include rejection of the Rayleigh line, focus light, disperse wavelengths, and present the light to a sensitive detector. LaPlant provides an excellent overview of lasers, spectrograph, and detectors [40]. Additional development and considerations of miniaturized instrumentation for handheld and portable Raman spectroscopy have been reviewed thoroughly by Crocombe [21]. There are additional instrument design considerations for installation in an industrial process environment including compatibility with hazardous, wash-down, or control room environments and integration with control systems or automation platforms. Since sampling by Raman can be achieved from the nanoscale to mega-production, this review will focus on these sampling platforms.

### Sampling at the nano- and micro-scale

Sampling versatility is a key advantage of Raman spectroscopy. Raman spectroscopy uses laser wavelengths in the visible and near-infrared, and it can be combined with inexpensive glass-based optical components like microscope objectives, mirrors, and lenses. Raman microscopy is typically employed to measure samples at the micron scale and even the nanometer scale with SERS and TERS techniques. The ability of Raman to use standard optical components allows easy integration of Raman equipment with standard microscopy equipment for microspectroscopy or imaging applications. Recent advances in Raman microscopy improve the utility for non-specialists and include automated depth profiling or focus adjustments, integration with multi-well plate readers, spectral libraries, rapid high-resolution imaging without manual adjustments, and particle analysis. The sampling and time requirements of Raman microscopy should not be overlooked when using multi-well plates. In a couple of Raman microscopy reports we review later in this paper, samples were deposited into 96-well plates and measured by a Raman microscope. Rapid evaporation in microtiter plates is an acknowledged issue [41, 42]. Moreover, since most commercial Raman microscopes employ epi-illumination geometry, a microtiter plate needs to be uncovered to collect Raman signals from the liquid in the well. Variable volume loss due to evaporation may account for any potential suboptimal model performance and impact model transferability. Volume loss from evaporation may be addressed using a Raman-compatible cover plate or by integrating robotic liquid handling platforms so that samples are automatically delivered to the well and then quickly analyzed by Raman microscopy.

### Sampling at the macro- and bulk scale

**Fig. 1:**
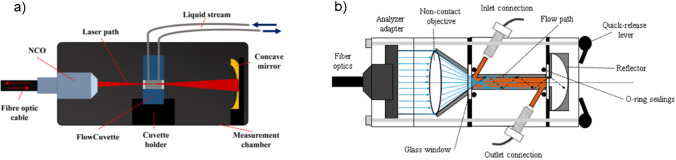
Schematic of Raman-based flow cell measurements. In one design (a), Raman signal was collected from a cross-section of a cuvette in which liquid was streamed. Within the measurement chamber, a non-contact optic was used to focus laser light into a cuvette and collect back-scattered Raman signal. Transmitted photons were focused back into the cuvette using a concave mirror on the opposite side of the cuvette from the non-contact optic. In another design (b), laser light was delivered into a measurement chamber by fiber optics. A fiber adaptor and non-contact objective were used to focus laser light into the flow path for measurements in a longitudinal direction. A reflector at the end of the flow path was used to focus light back into the flow path. In both cases, the fiber optic probe used a backscattered fiber geometry. Figure permissions: panel a was reused by permission of the publisher, John Wiley and Sons. Panel b was used under the open-access license. © 2019 by the authors. Licensee MDPI, Basel, Switzerland. This article is an open-access article distributed under the terms and conditions of the Creative Commons Attribution (CC BY) license

Measuring larger volumes is impractical using microscopy equipment. Handheld, flow cells, and fiber-optic probes are more typically used for these applications. Similar to assessing any analytical instrument for an application, the features of these sampling types should be balanced with its possible drawbacks. In the case of handhelds, the benefits of small size and portability can be understood in the context of reduced resolution, range, and sensitivity. For this reason, handheld Raman is most often used in materials identification. Handheld Raman provides sample identification of raw materials outside of the laboratory, often while they are still in their containers. A detailed discussion on handheld Raman spectroscopy is outside the scope of this paper, and interested readers can learn more on this topic with this excellent review by Crocombe [21]. For process or in-line sampling applications, fiber optic probes can be inserted directly in the process. Fiber-optic sampling probes are a key technology enabling process Raman spectroscopy for many reasons. One benefit of using sampling probes is that collecting a sample for laboratory analysis during industrial processes poses contamination and safety risks. In the chemical industry, for example, processes may involve toxic chemicals or hazardous conditions, increasing the safety risks of manual sampling. In upstream bioprocessing, manual sampling increases the risk of contaminating the sterile cell culture. Variations in the geometry of optical fibers and lenses within the probe can enable a variety of sampling options including backscattered, large volumetric, spatially offset Raman, and transmission Raman. Our 2016 paper described the variety of sampling probe geometries in more detail [18]. In addition to in situ probes previously described, flow cells provide a convenient interface for fiber optic probes to allow the measurement of low volume liquids or gases in constant flow. Flow cells are typically constructed from stainless steel and have three ports. Two ports are connected to the liquid or gas flow channels/tubing, and a third port is for the Raman probe connection. Figure 1 shows two types of flow cells, where the sample cell can be interrogated in a cross-section or longitudinal direction using a backscattered probe geometry.

In bioprocessing, there are variations from laboratory to process bioreactor equipment that impart two major requirements on probe design. The first requirement is that the probe housing needs to be compatible with the installation environment, process chemistry, and sterilization and cleaning protocols across multiple scales. Table 1 gives an overview of bioreactors, Raman probe equipment, and sterilization protocols for the scales often used in upstream bioprocessing. Miniature bioreactors provide a high-throughput approach to quickly optimize process conditions and cell engineering. These miniature bioreactors use small volumes and have typically not been amenable to spectroscopy measurements. Until recently, integration of the miniature bioreactor with Raman needed to be performed off-line without scale-specific probes or a unified software platform providing integrated pump, bioreactor, and Raman probe control [43]. Newly integrated at-line commercial systems address this gap [44]. Lab or process development scale bioreactors employ headplates to install sensors or spargers above the bioreactor’s impellor, and use autoclave to sterilize the equipped headplate prior to the culture. The immersion length of an in situ Raman probe may vary depending on the bioreactor size and working volume. A removable probe head ensures that the immersion materials are compatible with autoclave, and probe components not compatible with autoclave can be removed from the probe system. Pilot and manufacturing scale bioreactors use side ports to install sensors along the side of the impellor and use Clean in Place (CIP) and Sterilize in Place (SIP) procedures. Difference in cleaning/sterilization protocols, restriction on immersion length, and preventing personnel from inadvertently bumping are a few reasons that necessitate a probe specific for side-port installations. Increasingly, single-use bioreactors (SUBs) can be used in process development to manufacturing because they provide a convenient platform, reduce the cleaning burden, and eliminate cross-batch contamination. Raman probe compatibility with SUBs includes gamma sterilization and meeting material conformity standards. These various bioreactor types necessitate different probe exterior designs to meet the installation environment and user needs. How probes and analyzers are used in process development versus commercial manufacturing is an additional consideration for bioprocessing. Process development may use a single analyzer equipped with multiple probes for quick knowledge building and model development. Commercial manufacturing may use double redundant systems, where two probes from two analyzers monitor a single bioprocess.

**Table 1:** Overview of bioreactor equipment, Raman measurement probes, and sterilization/cleaning protocols used for the various scales used in upstream bioprocessing

Scale (typical volume range)	Bioreactor equipment	Raman probe equipment	Cleaning protocols
High-throughput (15μL–250 mL)	Micro- and miniature bioreactors	Flow cell + probe	Wash flow
Laboratory (1–20L)	Headplate bioreactor Shake flask Perfusion bags SUB (rigid plastic)	In situ probe Reusable + single-use component system	Autoclave Pre-sterilized
Pilot, clinical and commercial manufacturing (> 20L to thousands of L)	Sideport bioreactor SUB (bag based)	In situ probe(s) Reusable + single-use component system	CIP/SIP Gamma radiation (SUB)

Another requirement is that the fiber optic geometry design should be consistent to ensure good model transfer. Raman spectroscopy does not require a defined path length, which is an advantage over other vibrational spectroscopy techniques for measurements in turbid media. However, the turbidity of a cell culture or fermentation varies as cell density increases. Thus, fiber optic probes with a backscattered optical fiber geometry with a focus slightly away from the optical window have been shown to yield consistent and robust results across scales [45, 46]. Consistent design of the internal fiber optic geometry across fiber optic probes ensures cross-scale model transfer, and this aspect allows scalability to minimize the need to collect scale-specific data.

### Raman data analysis

Raman spectra are a “big data” source, as each spectrum is encoded with the sample’s chemical composition and molecular structure information. That encoded information is found through the Raman band position, width, height, and area. To extract useful sample or process information, Raman spectra can be analyzed by univariate or multivariate models to correlate changes in the Raman spectrum to known values. Univariate models are simple to use, as they use band area or intensity ratios or band center of gravity as the model data. This approach works well when there are sharp, well-resolved, and unique Raman bands in the spectrum. Spectra involving complex mixtures or samples, such as those from cell cultures, fermentations, or biological molecules, have broad and overlapping features. In those cases, multivariate models and chemometric practices are used. A comprehensive overview of multivariate data analysis (MVDA) techniques is beyond the scope of this review paper. The reader is referred to the “Chemometrics in Spectroscopy” book by Mark and Workman [47] for fundamental education and practical aspects of building chemometric models. We have seen more papers in the past few years that discuss obtaining good data model transferability and best practices for Raman-based MVDA in upstream bioprocessing.

While it may seem intuitive, an important step in model development is to obtain high-quality Raman data as well as high-quality data from the reference method. Hardware design impacts the quality of the Raman data, which in turn affects model specificity, robustness, and the ability to transfer or scale. Hardware built for high accuracy with a uniform internal design, but with housing configurable to the installation environment, has a few advantages including reproducible cross-instrument performance, compatibility with “turn-key” sampling probes, and ease of on-site servicing. This allows the user to perform calibration transfer from instrument to instrument and prevents calibration rework in the event of subsampling or a laser or detector change. Another important consideration is the choice of probe. Probe selection impacts representative sampling and model development, and certain probe optics may adversely affect model transferability or robustness in turbid media and solids [48, 49]. A thorough design of experiments will help to identify important parameters, develop a risk-based assessment of sources of spectral variance, and address colinear variables.

**Fig. 2:**
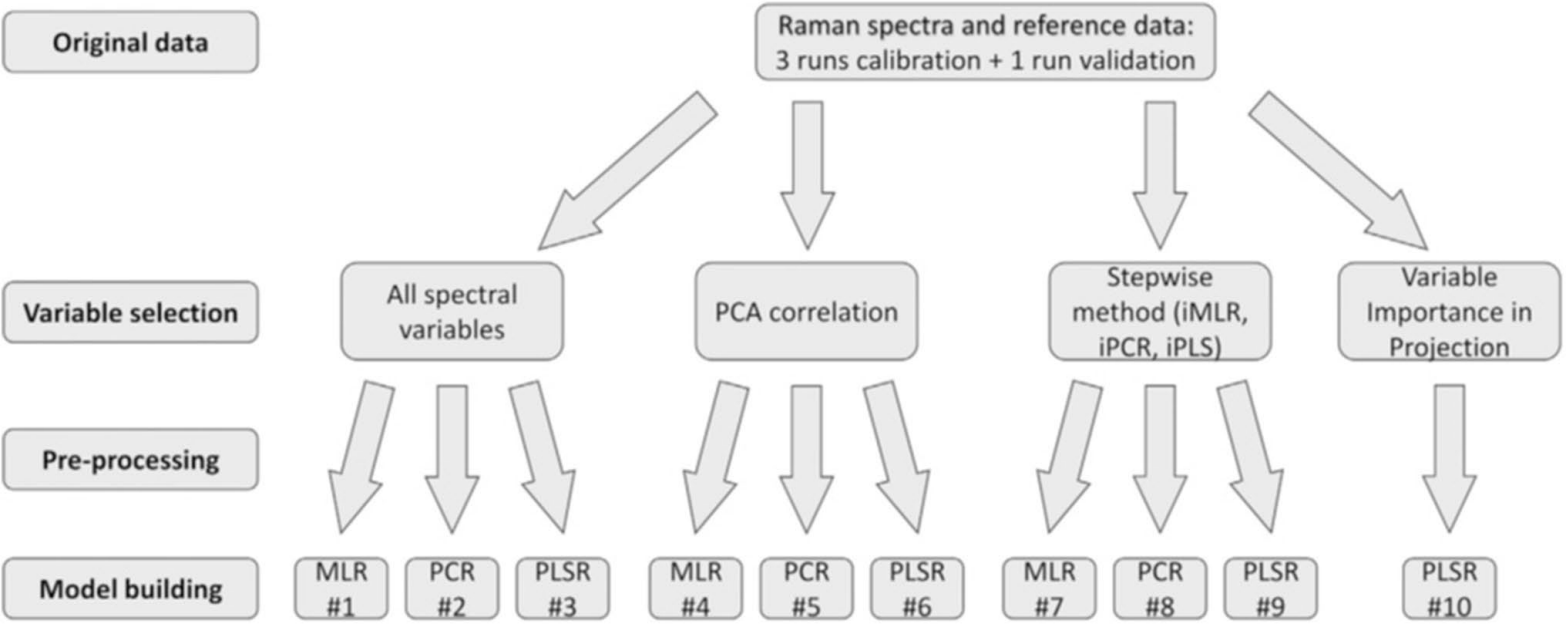
Example of experimental approach for Raman model development. Optimization of model performance is an iterative process involving data preprocessing, algorithm selection, calibration, and validation. While there are steps toward automating model development, it remains a manually intensive process. Raman data were first input into various methods for variable selection including principal components analysis (PCA), multiple linear regression (MLR), principal components regression (PCR), partial least-squares or projection to latent structures (PLS), or variable importance in projection (VIP). Data were then pre-processed using Savitzky-Golay first or second derivative, SNV, multiplicative scatter correction, and mean center or autoscale. MLR, PCR, and PLS regression were used to model data. Figure permissions: figure reused from Kozma et al. paper with permission from publisher, [51] © 2018 Elsevier B.V. All rights reserved

Another step in model development is data preprocessing. For a new process, finding the best combination of data preprocessing steps may be an iterative and manually intensive process. However, a review of preprocessing steps for upstream applications, shown in Table 3, indicates an industry consensus toward using 1^st^ or 2^nd^ Savitzky-Golay derivative, Savitzky-Golay smoothing (window size varies), standard normal variate (SNV) to correct for variations in optical scattering, and using a spectral window of ~ 400–1800 cm^−1^ and ~ 2500–3200 cm^−1^. While these preprocessing steps may be approaching standard use, optimization and automation of data preprocessing remain intensely studied. André et al. [50] describe the effect of acquisition time, natural variance within a batch and between batches, and variable selection on model optimization. Variable selection is a preprocessing step that is becoming more widely used, and several selection approaches have been reported [51, 52]. Figure 2 shows the experimental approach, including variable selection and pre-processing, to improve the quantification of glucose by Kozma et al. [51]. One approach, from Santos et al. [53], was to find a direct correlation of metabolite concentration with Raman bands through sample spiking of glucose, lactate, or glutamate. Extending these principles into a more automated model optimization approach or using a digital twin enables advanced process control and Industry 4.0 applications [54, 55]. The final steps in model development are algorithm selection, building, and validating the model. Modeling trends observed in the past few years of Raman spectroscopy in bioprocessing are an intensified emphasis on model transferability, extending models beyond partial least squares (PLS, also known as projection to latent structure) or principal components analysis (PCA), and integration of models into machine learning or artificial intelligence.

Important aspects of Raman bioprocessing models are scalability, transferability, and, to a varying degree, robustness against host cell lines, media types, and other process-related changes. The principles of cross-instrument model transferability were reviewed in 2018 by Workman [56]. Cross-instrument transferability also occurs when scaling from lab-to-process. Model scaling from lab-to-process is a common occurrence in biopharmaceutical development and an important consideration in the analytical lifecycle. Here, the ability of a Raman probe to provide consistent data no matter the scale is an important consideration. The differences between the laboratory and manufacturing environments necessitate different instruments with different housing designs to meet the demand of each of those environments. It is impractical to expect that a laboratory instrument will meet the physical demands of continuous operation in a wash-down or hazardous environment that are common in a manufacturing environment. Yet, it is not impractical to expect a laboratory-generated model to transfer to process equipment with minimal scale-specific data.

Thus, the model needs to not only be scalable from lab-to-process but also transferable across multiple instruments. Another growing consideration in bioprocess model development is cross-host transferability. These three aspects—scalability, instrument transferability, and cross-product transferability—comprise the phrase generic behavior. These aspects may complicate the model transfer process and further underscore the importance of high-resolution and high-quality data. There are several underlying motivations for generating models with generic behavior. The first motivation is to support GMP manufacturing sites which may produce various products using various media or cell lines. Another motivation is to quickly support clinical manufacturing that may only produce one or two batches of a therapeutic. Yet another motivation is a need to maximize the utility of the Raman data collected, since a lot of data is typically collected to account for the matrix complexity. The issue of model transferability was first addressed in 2015, with Berry et al. [45] reporting on cross-scale model transfer and Mehdizadeh et al. [57] reporting on cross-product transferability. Recent work has expanded on the principle of generic models, with a focus on improved predictions for multiple parameters and use in GMP manufacturing.

Creating a model independent of cell line for glucose, lactate, total cell concentration (TCC), viable cell concentration (VCC), glutamate, ammonium, and product concentration to support clinical or commercial manufacturing was the goal of a study by Webster et al. [58] in 2018. In that study, three lab-scale (5L) cultures were performed using CHOK1SV GS-KO cell lines, producing different mAbs. Cultures were monitored in-line by Raman and twice a day by off-line HPLC for product concentration and off-line biochemistry for metabolites and cell growth. Raman data were preprocessed to truncate the spectral region to 500–1700 cm^−1^, and a 1^st^ or 2^nd^ derivative, a Savitzky-Golay filter and SNV, was applied. A PLS model was developed for each parameter and the models were qualified against a new cell line to test generic behavior to new cell lines and a 10L culture to test generic behavior to culture scale. Table 2 shows the model figures of merit for each of the models. They found that most of the models were able to accurately track changes, with glutamate and product concentration being the exceptions. For those parameters, the authors hypothesized that the accuracy of the off-line measurement affected the glutamate model and collinearity with other cell-related parameters, and a lack of early-stage data affected the product concentration model. André et al. [59] extended the concept of cell line generic behavior into cross-species models, where they demonstrated the feasibility of model transfer for CHO (mammalian), HeLa (mammalian), and Sf9 (insect) cell cultures with the transfer of the glucose and lactate predictions into HEK-293 cell cultures.

**Table 2:** Model figures of merit for several process parameters assessed for a generic Raman model by Webster et al. [58] in 2018. RMSEP units are in the same units as the measured parameter. Except for the glutamate and product concentration models, the models were able to accurately predict parameters and were generic for new cell lines (I and II) and scale (III). [58] Adapted from reference [58]

Parameter	Range	*R*^2^_p_			RMSEP		
		**I**	**II**	**III**	**I**	**II**	**III**
Glucose (g/L)	0.44–10.12	0.99	0.98	0.99	0.47	0.43	0.41
Lactate (g/L)	0.00–3.76	0.96	0.97	0.94	0.30	0.22	0.18
Glutamate (mM)	0.00–5.34	0.60	0.18	0.56	0.97	1.63	0.89
Ammonium (g/L)	0.009–0.242	0.88	0.81	0.93	0.02	0.04	0.02
VCC (× 10^6^ cells/mL)	0.51–34.87	0.98	0.99	0.99	1.90	2.32	1.48
TCC (× 10^6^ cells/mL)	0.51–35.58	0.98	0.99	0.99	2.25	1.97	1.34
Product (g/L)	0.00–4.70	0.94	0.94	0.99	1.21	0.75	0.98

Using an entire dataset to predict a new measurement point is called a global model and, just like any model, it needs to balance robustness and accuracy. A different approach is to use only the local data, based on a similarity or distance approach, to predict a new measurement point. This approach is called a local model. Local models provide an attractive alternative to global models because they can dynamically respond to process conditions, account for process drift, and simplify model maintenance. Integrating just-in-time learning (JITL) to develop local generic models of glucose, lactate, glutamine, glutamate, calcium, sodium, viability, and viable cell density (VCD) was reported by Tulsyan et al. [60] in 2019. A library of 3800 off-line and Raman measurements were used to create a library. Notably, Raman data were preprocessed by only truncating spectra to remove the 100–300 cm^−1^, 1850–2900 cm^−1^, and 3200–3425 cm^−1^ regions and taking the log of the spectrum. When a new spectrum was introduced into the library, relevant data from the calibration set was determined on the Euclidean distance between the new data point and the calibration set. The relevant data were then used to build a local model, whether it was a PLS or a nonlinear Gaussian process (GP). The local JITL model was compared to a global model for all parameters. Use of the local JITL model produced improvements in model root mean square error (RMSE) ranging from 4.4% (sodium) to 44.89% (ammonium) for an independent validation of the PLS model and ranging from 9.2% (sodium) to 53.46% (ammonium) for an independent validation of the GP model. The approach was tested for scalability, new products, and cell lines with success. This method was then expanded by creating a dynamic library as new measurements were made available and integrated into nonlinear machine learning MVDA models for automated data time matching, model calibration, assessment, and maintenance [61]. Using data from multiple batches, where data are collected asynchronously, impacts the dataset linearity. One approach is to synchronize the data in software before applying multivariate analyses, and this approach is commonly used to time-sync chromatography peaks or pH, temperature, or dissolved oxygen. Time matching in-line and off-line data was automatically performed in the JITL studies by Tulysan and is also addressed by Liu et al. [62] using a different approach. In most cases, Raman data are collected in series over multiple bioreactors. Liu et al. [62] reported the application of correlation optimized warping (COW) on Raman data collected during multiple CHO cell cultures before using multiway PCA to define bioprocess control charts. This approach was successful in identifying batches within the control limits, and identifying batch contamination before traditional methods.

A comprehensive study, spanning 35 cultivations, was reported by Santos et al. [63] with the goal to understand the major risk factors on general and local Raman models. The 35 cultivations spanned 4 CHO cell lines, 8 different clones, 4 scales (2L, 7L, 15L, and 10,000L), and two cultivation modes (fed-batch and perfusion). The cultivations were monitored by in-line Raman spectroscopy, pH, temperature, and dissolved oxygen with a daily off-line measurement for glucose, lactate, and titer. Raman data were modeled using a general PLS model from all 35 cultivations, and two local models. The excluded local level model built a local model guided by batch conditions with a leave-one-out validation, and the single level local used only spectra corresponding to a single level. The results from the paper were three-fold: perform an initial risk assessment, evaluate the performance of a general versus local model, and propose an approach to reevaluate risk assessment. An interdisciplinary team of analytical method development and mammalian cell culture experts performed a risk assessment, based on their prior knowledge and evaluation of local models, to understand the risks to obtaining optimal cultivation conditions of high titer, yield, and viable cell density. Multiple risks were identified in materials, environment, process, measurement, instrument, and manpower. However, the materials category was the most important since it directly impacts design space and influences the chemical matrix of the cultivation. After FMEA, eight major risks were initially identified: scale, base powder, reference method errors, cultivation mode, final media composition, main feed, clone, cell line, media lot, and temperature. After analyzing the data, the risk factors were reassessed, and scale and base powder remained the factor with the highest risk priority number (RPN). Clone and main feed increased their RPN, while cultivation mode and final media composition decreased their RPN. With respect to model scalability, they found that the glucose, lactate, and protein models could transfer from small to large batches using only scale-specific data. However, they suggested that scale-specific data could be collected as a precaution and it adds to the process knowledge. Identifying risks, using process knowledge and data, then reassessing those risks is a cornerstone of the PAT and QbD initiative. This paper provides an excellent example of putting those principles into practice and it can serve as the basis to establish Raman PAT operation guidelines.

## Applications in biopharmaceutical manufacturing

### Basic science in cell and molecular biology

Raman spectroscopy is a powerful technique to study biological cells, fluids, and tissues [64]. Extension of Raman techniques developed for biomedical and cell biology applications to industrial bioprocessing has brought a new understanding of mammalian cell metabolism and media chemical composition. In particular, we are pleased to see new research papers on using Raman microscopy in the laboratory to understand CHO cell metabolism and identify highly producing cells during cell line optimization [65, 66], identify different stages of cell death [67], and monitor media components under stressed UV or heat conditions [68]. While the research cited was performed in an academic laboratory, they establish feasibility for industrially relevant applications including verification of transfection, improved understanding of cellular death mechanisms, and identification of high-producing cells.

### Upstream monitoring and control

Foundational work in 2011 to 2016 quickly established industrial use of Raman spectroscopy for upstream monitoring and control [69]. Raman’s specificity and ability to measure aqueous systems in situ translated to industrial benefits of rapid model development, model scalability, and automated control of multiple biochemical parameters. Since 2016, more reports on Raman-based bioprocess control establish Raman spectroscopy as a leading PAT for in-line glucose monitoring and control. In these newer reports, we see monitoring and control of additional parameters including lactate, amino acids, and cell attributes. Table 3 shows an overview of papers since 2016 in Raman-based upstream monitoring and control. While most of upstream Raman papers discuss in-line Raman spectroscopy, collected directly in the bioreactor, there have been a few reports using Raman microscopy. These include a Raman microscope coupled to an automated plate reader to support early-stage scale-down conditions [70], an academic study that used in-line viscosity and off-line Raman measurements as input into a Monad model of CHO cell metabolism, [71] and an off-line time-gated Raman/SERS microscopy approach coupled to a custom microwell plate for *E. coli* fermentations [20]. Additional work in the laboratory, using shake-flask or lab-scale bioreactors, compares the functionality of Raman and NIR for measuring individual parameters such as glucose, lactate, cell viability, ammonium, and glutamine [46, 72]. In-line Raman studies described below further establish the use of Raman for measuring major biochemical process parameters such as glucose, lactate, glutamine, and glutamate. These recent studies also expand the utility of Raman into measuring other parameters such as amino acids, pH, cell viability, and cell volume.

**Table 3:** Overview of Raman spectroscopy papers in upstream mammalian cell bioprocessing monitoring and control applications since 2016. Glucose is an important parameter that is measurable by in-line Raman. Additional parameters tested are amino acids, cell-related parameters, protein product, and pH-influencing molecules. Although a variety of Raman data preprocessing techniques were reported, there appears to be a consensus that a combination of the first or second derivative, SNV, and spectral region selection is suitable for real-time monitoring and control applications. Guide to the preprocessing techniques: (1) cosmic ray removal; (2) intensity correction; (3) variable or spectral region selection; (4) Savitzky-Golay 1st or 2nd derivative; (5) multiplicative scatter correction (MSC); (6) standard normal variate (SNV); (7) autoscale and mean centering; (8) Savitzky-Golay smooth; (9) baseline correction. There are many candidate model figures of merit to report, and we standardized on reporting the concentration range and root mean square error of prediction (RMSEP). The authors provide an estimated range noted as ( ~) based on the paper’s figures if a range was not explicitly provided in the paper’s text. Since many of the papers report on iterative model development with several developed models, a range of model parameter values are included in the table if they were in the paper. The reader is encouraged to refer to the individual papers to learn how each study optimized the model according to the specific application needs

Raman-measured parameter(s)	Cell line Target molecule	Preprocessing techniques	Model(s) used	Model figures of merit
Glucose [75]	CHO DG44 mAb	4, 3, 6	PLS	Range: 1.02–14.46 g/L RMSEP = 0.24 g/L
Glucose [80]	CHO DG44 Adalimumab biosimilar	9, 6, 7	PLS	Range: 0–70 mM RMSEP = 5.2 mM
Glucose [46]	CHO IgG1	1–7	PLS	Shake flask Range: 0–60 mM RMSEP: 1.3797 mM
				10 L Range: 0–60 mM RMSEP: 4.0297 mM
				100 L Range: 0–60 mM RMSEP: 4.0453 mM
pH [76]	CHO mAb	3, 4, 6	PLS	pH Range: ~ 6.6–7.3 RMSEP (full range): 0.066–0.076 RMSEP: 0–4 days: 0.020–0.039; days 4 + : 0.034–0.039
				pH from lactate + pCO_2_ Range: ~ 6.6–7.3 RMSEP: 0–4 days: 0.019–0.036; days 4 + : 0.030–0.034
Glucose Phenylalanine [73]	CHOK1SV GS-KO® mAb	3, 4, 6	PLS	Glucose Range: ~ 0–11 g/L RMSEP: 0.42 g/L
				Phenylalanine Range: ~ 20–580 mg/L RMSEP: 21.3 mg/L
Glucose Lactate Ammonia[52]	CHO mAb	3, 4, 6, 8	Support vector machine radial, random forest, Cubist, PLS	Glucose Range: 5–25 mM RMSEP: 1.437 mM
				Lactate: Range: 0–30 mM RMSEP: 2.0 mM
				Ammonium Range: 0–9 mM RMSEP: 0.819 mM
Glucose Lactate Antibody VCD [70]	CHO Antibody-peptide fusion protein; modified IgG1	9, 8, 6	PLS	Glucose Range: 1–5 g/L RMSEP: 0.38 g/L
				Lactate Range: 0–12 g/L RMSEP: 1.16 g/L
				Antibody Range 0–2 g/L RMSEP: 0.09 g/L
				VCD Range: 0–40 × 10^6^ cells/mL RMSEP 3.49 × 10^6^ cells/mL
Glucose Lactate Antibody VCD Glutamine Ammonium [72]	CHO DG44 anti-Rhesus D antibody	3, 6, 7	PLS	Glucose Range: 0–25 mM RMSEP: 1–1.04 mM
				Lactate Range: 0–20 mM RMSEP: 2.38–2.51 mM
				Antibody Range: 0–0.4 g/L RMSEP: 0.02 g/L
				VCD Range: 0–80 × 10^5^ cells/mL RMSEP: 5.31 × 10^5^ cells/mL
				Glutamine Range: 0*–*3 mM RMSEP: 0.42–0.44 mM
				Ammonium Range: 1–5 mM RMSEP: 0.76–0.77 mM
Tryptophan Tyrosine Phenylalanine Methionine [74]	CHO mAb	4, 6, 3, 8	PLS	Tyrosine Range: 0.28–4.05 mM RMSEP: 0.35 mM
				Tryptophan Range: 0.29–1.81 mM RMSEP: 0.07 mM
				Phenylalanine Range: 1.23–3.05 mM RMSEP: 0.32 mM
				Methionine Range: 1.70–2.50 mM RMSEP: 0.68 mM
Capacitance Viable cell density Viability Average cell diameter Viable cell volume [77]	CHO mAb	3, 4, 6	PLS	Capacitance RMSEP, full range: 1.54 pf/cm RMSEP, combined slope: 1.40 pf/cm
				VCD RMSEP, full range: 1.20 (10^6^ cells/mL) RMSEP, combined slope: 1.05 (10^6^ cells/mL)
				Viability RMSEP, full range: 0.58% RMSEP, combined slope: 0.40%
				VCV RMSEP, full range: 6.15 E + 03 (μm^3^/10^6^ cells/mL) RMSEP, combined slope: 6.75 E + 03 (μm^3^/10^6^ cells/mL)
				Cell diameter RMSEP, full range: 0.69 μm RMSEP, combined slope: 0.58 μm
Glucose Lactate Glutamate Glutamine [78]	T-cells from human donors	1,3,4,6	PLS and univariate	Glucose Range: ~ 0–4 g/L *R*: 0.987
				Lactate Range: ~ 0–3.5 g/L *R*: 0.986
				Glutamate Range: ~ 0.05–0.2 g/L *R*: 0.829
				Glutamine Range: ~ 0–1 g/L *R*: 0.922

The success of Raman-based glucose feed strategies supports extending the technique to amino acid feed strategies. Raman-based automated glucose and phenylalanine feed was demonstrated by Webster et al. [73]. Manual-based feed control and automated feed control were performed on a fed-batch culture with two different CHOK1SV GS-KO® cell lines. Phenylalanine was selected as a target amino acid to control with Raman because of its importance in preventing tyrosine incorporation into the primary structure of a mAb. To build the predictive model, Raman spectra were collected in-line throughout the culture duration and compared against reference glucose and amino acid measurements. Off-line measurements of glucose, amino acid, osmolality, and viable cell density were collected daily and product CQA’s of glycosylation and charge variant were collected from day 4 until harvest. Raman estimations of glucose and phenylalanine were input into a PID algorithm which controlled the feed pumps. The prediction model parameters Q2, RMSEE, and RMSECV all indicated that the model did not overfit the data. The model RMSEP, 0.42 mL/L for glucose, and 21.3 mg/L for phenylalanine were similar to other reported RMSEP values for similar Raman models. The authors demonstrated that the automated feeding approach had resulted in less variance in the off-line profiles including VCC and viability. Both glucose and phenylalanine were better controlled with automated versus manual control. Most amino acid profiles were also more consistent with the automated control and there was no evidence of amino acid exhaustion or accumulation as a result of the automation. The new control strategy had minimal effect on VCC, viability, glycosylation, and charge variance. The authors did observe that the product concentration from the automated control runs was ~ 20% higher than the manual control runs. This promising study demonstrated that a Raman-based automated feed strategy for glucose and an important amino acid did not adversely affect product quality and resulted in a more consistent process with yield improvements.

Establishing the feasibility of Raman-based quantification of amino acids to replace off-line HPLC analysis was the goal of a study by Bhatia et al. [74]. Tryptophan, tyrosine, phenylalanine, and methionine were selected for Raman analysis because of their importance to cell growth conditions. The authors hypothesized that control of these amino acids should result in higher productivity and more consistent product quality. Off-line Raman measurements of amino acid stock solutions were used to identify unique spectral regions for each of the four amino acids. In-line Raman spectra were collected from seven batches and used to calibrate a PLS model, while samples from the lab and pilot scale were used for model validation. UPLC analysis of the supernatant from daily off-line samples provided a reference measurement. The PLS models performed well for tryptophan, tyrosine, and phenylalanine with indications that the model was robust and stable. The model for methionine did not perform as well as the other three models, and the authors hypothesized that the relatively weak Raman signal of methionine and overlapping bands with cysteine were contributing factors in the model performance. Overall, the Bhatia et al. [74] and Webster et al. [73] studies establish the feasibility of Raman spectroscopy to quantify amino acids in-line during a cell culture bioprocess. These studies support the continuation of this research with the eventual goal of automated amino acid monitoring and control.

**Fig. 3:**
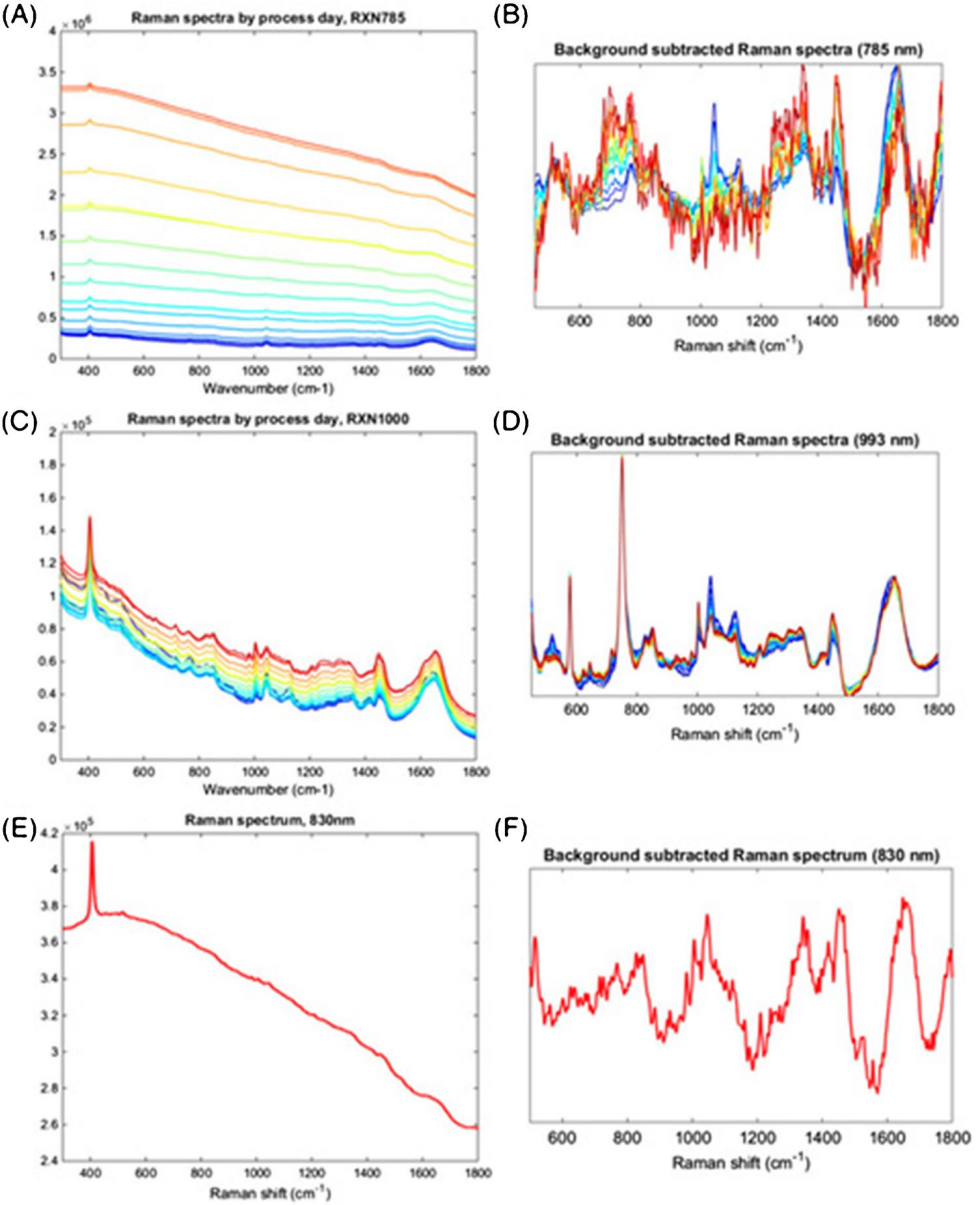
The effect of laser wavelength on Raman spectra throughout a high-density CHO cell culture. The initial use of 785 nm excitation (panels A and B) resulted in the observation of late-stage fluorescence which prevented accurate prediction of glucose after the third day of the culture. Using higher laser wavelengths at 830 nm (panels E and F) and 993 nm (panels C and D) decreased fluorescence. Using the 993 nm wavelength, which provided the most fluorescence reduction, enabled Raman-based glucose control throughout the duration of the culture. Figure permissions: figure reused from Matthews et al. paper with permission from publisher, John Wiley and Sons (75)

Raman-based monitoring and control of process parameters may enable process intensification strategies, even in the presence of strong fluorescence [75]. In 2018, Matthews et al. [75] reported observing high amounts of fluorescence after the third day of a high-density DG44 CHO culture that prohibited Raman-based glucose quantification when using the standard 785 nm Raman analyzer. This unexpected observation led to a post hoc analysis of 14 other cell cultures from a variety of cell lines and process conditions to identify possible sources of fluorescence. That post hoc analysis showed that the media or feeds were not contributing to fluorescence, and it was hypothesized that the accumulation of an unknown parameter associated with the cells was responsible for the fluorescence. The most cost-effective approach was to use a Raman system operating at a higher wavelength (*λ* = 993 nm). Figure 3 shows a comparison of 785 nm, 830 nm, and 993 nm excitation of the bioprocess with time. Raman spectra obtained with a 993 nm system reduced autofluorescence to enable glucose quantification and automated feeding strategy.

A series of three papers by Rafferty et al. [76, 77] in 2020 researched avenues to expand the functionality of in-line Raman. In one study, in-line Raman was compared to in-line pH, off-line pH, off-line lactate, and carbon dioxide partial pressure (pCO_2_) to determine if Raman spectroscopy could provide secondary pH measurements in two CHO cell lines. pH is a critical process parameter, but the drift of in-line pH meters requires daily off-line confirmation and the daily sampling poses a contamination risk. The authors hypothesized that changes to pH-influencing molecules are implicit in the Raman spectra, and Raman-predicted lactate and pCO_2_ values could be used to predict off-line pH. Raman spectra were first compared to off-line pH values across the entire 17-day culture, but they found that full-scale pH was too complex to model. To reduce the model complexity of the full-length data set, the authors segmented the culture data into an early stage and a late stage to generate an early and a late model. This approach slightly improved the model predictions, but there were prediction errors that were not consistent between the two cell lines. For example, there were model switching errors in cell line A that were not observed in cell line B and there were underpredictions in cell line B on days 9–11 that were not observed in cell line A. In another approach, the authors generated two off-line pH models from lactate and pCO_2_ values. The first model was based on off-line lactate and pCO_2_ values and the second model was based on in-line Raman-based lactate and pCO_2_ values. The model complexity was reduced by incorporating off-line parameter data. These first results support further model development for an overall goal of sampling-free bioprocesses.

A follow-on study by Rafferty et al. [52] had explored the use of Raman spectroscopy to support feeding strategies based on cell health, as measured by capacitance. Feeding strategies can follow a purely component concentration basis or a more holistic cell health approach. Examples of a component-based feed strategy are to measure glucose or specific amino acids, and then add those components to the media when the concentrations fall below a set point. The studies described in our 2016 paper and many papers listed in Table 3 follow this component-based feeding and show that Raman spectroscopy supports automated feeding strategies for single or multiple components. The hypothesis behind feeding strategies based on cell health is that more feed components can be tailored to optimize cell metabolism and yield. Capacitance measures important cell parameters including VCD, viability, and viable cell diameter. However, the technique is affected by physiological conditions and signal interference from non-viable cells. Moreover, there are two redundancies to the primary in-line capacitance probe in production: a secondary in-line probe and off-line VCD measurements. The authors tested two hypotheses: (1) that Raman could act as a possible redundant system for capacitance and (2) that Raman could act as the input for capacitance-based feeding strategies. The study was performed in eight production-scale cultures of CHO cells producing monoclonal antibodies. In-line capacitance and Raman were collected throughout the 13-day culture. Daily off-line VCD, cell diameter, and viability were also collected. Off-line data or Raman data were used to calculate VCD and viable cell diameter. In the feed study, a proprietary feed was used consisting of the complex nutrient media, glucose feed, and amino acid supplement. There were two approaches to modeling: a single model for the entire time course, and four linear models to reflect four stages. Six batches were used to develop the model, and two batches to test the model performance. The full and staged models were applied to the prediction of capacitance, VCD, viability, and cell diameter. The authors discussed the full and staged model performance and how model switching or dilution affected overall performance. The comparison of capacitance and Raman-based feed volumes showed that days 0–2 were outside the action range, likely because of the low cell concentration and narrow range. After day 3, Raman-based feed volumes were within 6% or less of capacitance-based feed volumes. A combined PAT approach using both capacitance and Raman reduces the dependence on one measurement type for complex feed strategies which could support other applications such as inoculation processes. Finally, Rafferty et al. [52] assessed the performance of support vector machines, random forests, and Cubist to predict glucose, lactate, and ammonium and compared those results from a PLS model. Data were collected from three bioreactor scales, 1L, 2L, and 2000L from two CHO cell lines. For glucose, lactate, and ammonium, the Cubist model slightly outperformed the PLS model, indicating that non-linear tree-based models could be applied to bioprocesses.

These recent upstream papers show an increasingly sophisticated use of Raman spectroscopy for advanced applications such as feedback-based process control, automation, integration of sensors and mechanistic knowledge, and efficient model optimization. Successes in mAb manufacturing form the application and economic basis for extending Raman into other platforms, including cell therapies. Baradez et al. [78] showed success in Raman-based monitoring of glucose, lactate, glutamate, glutamine, and ammonium during the cultivation of T-cells derived from human donors. The growing role of PAT in bioprocessing is supported by industry, regulatory bodies, and collaborative working groups as noted in an earlier section of this paper. With this increased process of information from many sources comes the challenge of data aggregation, visualization, and analysis. A roadmap to integrate PAT into automated knowledge extraction can bring comprehensive process understanding, support agile process development, and bring real-time release and Industry 4.0 principles to manufacturing. A roadmap proposed by Wasalathanthri et al. [79] called for identifying critical quality attributes (CQAs) and critical process parameters (CPPs), understanding the required precision and sensitivity for the measurement, and then make a risk-based assessment of PAT tools to decide when and how to measure CQAs and CPPs. As noted in an earlier section, Raman spectroscopy is a big data source, and the data are supporting advanced control strategies as well as an Industry 4.0 approach to bioprocess monitoring and control. Compatibility with multivariate analyses, automation platforms, redundant systems, and automated system checks enable scalability from development to commercial manufacturing. These reports since 2016 have shown that Raman spectroscopy is a proven and increasingly valuable PAT for agile process development and supports Industry 4.0 manufacturing initiatives.

### Downstream monitoring

Success in upstream monitoring applications and a deeper knowledge of Raman’s capabilities support the extension of Raman spectroscopy to downstream bioprocessing. Major benefits of Raman in downstream are a highly specific measurement on protein quality in a highly concentrated solution, and the same analytical technology used for upstream can be translated to downstream in a continuous bioprocess. However, it should be noted that publications in downstream have been limited until recently. One possible reason is the relative speed in which downstream operations are performed, which impacted cycle time and limited the acquisition time in which spectra could be collected using standard immersion probes. Another possible reason is the difficulty in finding an application in which Raman provides a cost-effective benefit over existing measurements such as UV–Vis and then proving out the technology. Despite these challenges, there have been many conference presentations since 2010 covering Raman of aggregation, release testing of buffers, filtration, and chromatography. We are now starting to see more original journal papers on the topic.

Starting in 2019, a series of papers described Raman applications to quantify the target molecule during harvest and monitoring quality attributes such as aggregation or monomer purity. New approaches to sampling and signal enhancement have begun to address technical challenges to integrating Raman into downstream bioprocessing. Flow cells for in-line measurements and multi-well plates for at-line measurements were employed with success. A Raman-integrated flow cell was described by Yilmaz et al. [81] for the purpose of quantifying immunoglobulin G (IgG) concentrations directly in permeate without sample removal [81]. Intensifying downstream activities using continuous manufacturing approaches, especially during perfusion-based harvests, represents a challenge because of the high volumes and low protein concentrations. A single-use bioreactor, equipped with a single-use perfusion device, was used to cultivate a 100L CHO cell culture producing different mAbs from three different CHO cell lines. A Raman flow well was integrated into the permeate line and in-line spectra were collected every 15 min during the last stage of the 15-day cultivation, the low cell-specific perfusion rate (CSPR) stage of growth. Additional off-line measurements were collected at the second cultivation stage, called the high-end pH-controlled perfusion (HIPCOP) stage. Ultra-performance liquid chromatography was the reference measurement. Data were pre-processed, after optimizing the approach, using a first derivative, 2^nd^ order polynomial with a 25-point fitting window and normalization using standard normal variate (SNV) to correct for a variable path length caused by differences in optical scattering. The Raman fingerprint region has many areas for relevant variable selection including the 900–1150 cm^−1^ backbone region, amide III envelope at 1230–1340 cm^−1^, CH_2_ deformation band at ~ 1447 cm^−1^, amide II at ~ 1551 cm^−1^, and amide I envelope at 1620–1700 cm^−1^. The model was developed using 371 samples of two IgG subclasses in the 0–6.87 g/L concentration range, and tested on 230 samples for 3 IgG subclasses. The model was then tested to unknown spectra to test generic behavior. The root mean square error of prediction (RMSEP) of 0.18 g/L indicated that the model could be transferred across IgG subclasses. The success of these experiments justified transitioning to an in-line Raman system, eliminating the need for off-line sampling.

Feidl et al. published two papers in 2019 on the use of Raman spectroscopy integrated with a flow cell to measure IgG during harvest [82, 83]. The goal of the first reported study was to establish the feasibility of the use of a custom flow cell integrated with Raman spectroscopy for in-line measurement of mAb concentrations during harvest with a high amount of impurities [83]. Off-line data sets were generated from cell culture supernatant pools collected from a CHO cell perfusion culture, in-line data sets were generated from breakthrough runs, and HPLC was used as the reference measurement. Raman spectra were collected for 30 s, corresponding to 0.25 column volumes with a 2-min residence time. After data preprocessing, the spectral ranges of 450–1820 cm^−1^, 1880–2530 cm^−1^, and 2590–3100 cm^−1^ were identified as the informative regions. A calibration model was developed from four breakthrough runs, and the model was tested using a rotation of the calibration sets in and out of the model, essentially a leave-one-run-out approach, which resulted in 1080 developed models. This approach had the added benefit of a preliminary assessment of model generic behavior and assessment of breakthrough run similarities. Optimization of the model root mean square error of cross-validation (RMSECV) was performed using decision tree analysis. An interesting result from the decision tree analysis was that the model performed well without needing to identify specific Raman bands, apply a derivative to the spectra, or use outlier removal tools. Applying the model of off-line measurements showed an improvement to the model robustness with respect to flow rate and higher titer concentrations. The optimized model showed good estimation with an average RMSEP of 0.12 mg/mL within 0–2.82 mg/mL and an average limit of detection of 0.24 mg/mL.

The next generation of flow cell design and a hybrid modeling approach for low mAb concentration monitoring was reported in another 2019 paper by Feidl et al. [82] A comparison of the developed flow cells is shown in Fig. 1. The first reported flow cell had built-in Raman analysis on the cell’s cross section (left), and the latter was designed for Raman measurements longitudinal to the flow cell. For this study, 2 off-line supernatant pools and 15 in-line breakthrough runs with concentration ranges of 0.3–0.6 mg/mL were measured by Raman. Raman signal was collected for 30 s. Raman data were calibrated using a partial least squares model, and the chromatographic process was modeled using a lumped kinetic model (LKM). Extended Kalman filtering (EKF) was used to combine process knowledge with in-line measurements. While Raman-PLS model had resulted in a reasonable model performance for the data rotations (RMSECV range 0.04–0.042, *R*^2^ range 0.7–0.86), and captured the trend of the breakthrough curve. However, there was also prediction scatter around the reference values. Lumped kinetic model was shown to have a good ability to predict the shape of the breakthrough run curve. EKF was applied to the Raman-PLS and LKM models to weight the relative input of the data model compared to the mechanistic model. The filter resulted in a more accurate mAb quantification, especially at the incipient breakthrough region.

Rapid at-line measurements may be used to support scale-down experiments. In 2020, Goldrick et al. [70] described the use of a standard Raman microscope for at-line measurements in a 96-well plate to support scale-down upstream and downstream studies. Raman microscopy is used to provide spatial and chemical information on a material on the microscopic level. A Raman microscope can also be integrated with microtiter plates for automated higher throughput analysis of small volume samples (< 1 mL). The upstream portion of the study was to assess Raman microscopy for glucose, lactate, viable cell density, and viability; the downstream portion of the study was to assess Raman microscopy for measuring total concentration and aggregation or fragmentation of a Fc-fusion protein during cation exchange chromatography purification. The study did establish the feasibility of integrating Raman microscopy with standard scale-down bioreactors and autosamplers for at-line measurements of small volumes using standard polypropylene and custom stainless steel 96 well plates. Scale-down and high-throughput applications may also be addressed using integrated Raman and miniaturized bioreactor systems that employ a flow-cell for low volume handling without evaporation [44].

### Product formulation

Given the benefits of Raman in protein analysis, the establishment of Raman as a leading PAT in upstream, and newly reported applications in downstream, it is not surprising to see an intensified interest in applying Raman spectroscopy throughout a biopharmaceutical product’s lifecycle. Emerging industrial biopharmaceutical applications of Raman spectroscopy include protein crystallization, aggregation, higher-order structure, and post-translational modifications. These aspects of Raman support many phases of product formulation from first-principles molecular knowledge to product release. Successful product formulation is an art and science. The science of biopharmaceutical product formulation includes knowledge of protein-solvent interactions, degradation pathways, molecule conformation, biological activity, metabolic pathways, and immunogenicity. Successfully navigating these interconnected aspects, as well as packaging and cost considerations, can be considered an art form. Reviews by Lee [84] and Parkins and Lashmar [85] provide a solid foundation for thermodynamic principles in the stabilization of proteins by co-solvents, degradation mechanisms, and analysis strategies. Importantly, they drew upon established biophysics principles and advocated for a “toolbox” approach to analysis rather than heavy reliance on a single analysis technique. Biophysical techniques can be extended to protein formulation studies to support accelerated stability studies, crystallization experiments, and understand protein-excipient interactions [86]. Raman spectroscopy has been used to support formulation development, with applications in aggregation, particulates, and real-time release of formulation buffers [87–89]. Two studies are highlighted to demonstrate basic science and quality control uses of Raman spectroscopy. In the first study, Raman microscopy was used to understand the role of cryoprotectants, glycerol, and trehalose, on lysozyme stabilization during freeze-drying [90]. In the second application, Raman spectroscopy has been used since 2006 to provide analytical quality control of compounded formulations stored in vials or directly through polymeric infusion pumps in hospital settings [91]. These studies support the extension of Raman spectroscopy into therapeutic protein formulation for improved understanding and quality control applications.

### Higher-order structure

One challenge in developing biopharmaceuticals is consistent product quality. Using the adage “structure determines function” from general biochemistry class, it is logical that the molecular structure of a biopharmaceutical has an important role in its efficacy, toxicology, and immunogenicity. Thus, higher-order structure is a critical parameter to monitor and control. PAT enables measurement of physical instabilities such as aggregation or chemical alterations including post-translational modifications or cross-linking which can complement stabilization formulation strategies [92]. Recent reviews highlight the many established and emerging biophysical tools available for higher-order structure analysis [93, 94]. It is curious that Raman is mentioned as an emerging technology in recent reviews of higher-order structure analysis tools because Raman has been used for nearly 90 years in biophysical measurements! Raman spectroscopy is an established analytical technique for examining biological macromolecules, protein structure, and dynamics [95]. As a measurement technique for proteins, Raman imparts many benefits. Raman spectra report on protein backbone and side-chain groups which can be used to characterize higher-order structure including the presence of α-helix, β-sheet, and random coil structures. Raman can measure proteins in aqueous or deuterated solutions, enabling the study of proteins in their solid or native state and in deuterium exchange studies. Minimal sample preparation, no requirements for exogenous labels, non-destructive nature, and rapidity of a Raman measurement means that the same protein sample can also be measured by other analytical techniques such as analytical ultracentrifugation. These well-known features have been harnessed in biology and biomedical fields to study tissues, cells, and biofluids [64].

A review of the historical literature establishes Raman as an important biophysical analysis tool. Biophysical studies of amino acids, synthetic polypeptides, proteins, and glycoproteins using Raman spectroscopy were reported as early as 1936. Early Raman studies complemented the known suite of biophysical analyses including crystallography, X-ray diffraction, neutron diffraction, microscopy, circular dichroism, isotope exchange, and infrared spectroscopy. Edsall [96–100] applied Raman spectroscopy to known biophysical techniques and published a series of studies starting in the late 1930s on “Raman Spectra of Amino Acids and Related Compounds.” These studies establish band assignments and band polarizability of native, ionized, substituted, and deuterated molecules, and the ability of Raman to elucidate the conformation of amino acids including the ability to distinguish backbone structure from side chains. By the 1970s, lasers made measurement times of large biomacromolecules more practical. From 1970–1976, Lord and Yu [101, 102], Bellocq et al. [103], Chen et al. [104, 105], Chen and Lord [106, 108], and Chen et al. [107] published a series of eight papers on “Laser-excited Raman of biomolecules,” focusing on proteins including lysozyme, bovine serum albumin, and beta-lactoglobulin. Importantly, these studies measured protein conformation under normal and denaturating conditions and used synthetic polypeptides as models of secondary structures such as α-helix or β-sheet. With this basis, Raman was applied to understanding larger biomolecules and enabled in-depth studies of particular groups within a small biomolecule for polypeptides, carbohydrates, or nucleic acids.[109–111] An extraordinarily useful review “Raman Spectroscopy of Biological Molecules” was published in 1972 by Koenig [112], and is essential reading because it provides a wealth of information about higher-order structure of amino acids, polypeptides, nucleic acids, proteins, and carbohydrates.

Raman and UV-resonance Raman spectroscopy are now established biophysical tools that complement other analyses in the PAT toolbox to understand protein higher-order structure [113]. They are starting to be used in structural analysis of therapeutic proteins because they can measure in the solution phase, are compatible with isotope exchange, are non-destructive, and provide a direct measurement of protein structure. An emerging technique for mAb analysis, UV-resonance Raman, and deep-UV resonance Raman are powerful methods for protein structural analysis. Its ability to selectively probe amide π-π* vibrations allows for simultaneous analysis of amide I, II, and III bands and C_α_-H amide bending for a direct protein secondary structure analysis [31]. Two recent papers from the FDA’s CDER Division of Pharmaceutical Analysis, Office of Testing and Research demonstrate deep-UV resonance Raman for understanding mAb structure under normal and stressed conditions [114, 115].

Spontaneous Raman has been used since the 1930s to understand protein structure, and more recently to understand, monitor, or control therapeutic protein structure. Isotope exchange studies have been used with Raman spectroscopy since Edsall’s studies in the 1930s and in the 1990s with mass spectrometry [116] Hydrogen deuterium exchange (HDX) studies are performed in deuterated solutions, where deuterium replaces exposed amide hydrogens and the hydrogen-bond network is disrupted. Spectral analysis of band position, center of gravity, or intensity can reveal global structural changes, local hydrophobicity of side chains, or exchange kinetics. Lysozyme is a well-characterized and thoroughly studied protein, and many of the studies described below used lysozyme as a model protein [117]. Lysozyme has been used as a model protein in biophysical Raman spectroscopy since 1970, and it remains a commonly used model protein for modern crystallography, higher-order structure, aggregation, glycation, and formulation studies [101]. Miura et al. [118] performed stepwise deuteration of lysozyme and α-lactalbumin to understand the local tryptophan (Trp) environment. Raman bands showed changes upon stepwise deuteration, where the 880 cm^−1^ band was sensitive to Trp H-bonding and the 1360 cm^−1^ band was a marker for local hydrophobicity. Comparison of Raman data with X-ray diffraction of lysozyme showed that four Trp sites in solution state were identical to the crystal structures, with different H-bonding at two Trp sites (Trp-28 and -111) between the solution and crystal states. In α-lactalbumin, the differences in two Trp sites (Trp-28 and -108) were greatly different from similarly located Trp sites in lysozyme. The importance of Trp in mAb higher-order structure was noted in a recent study by Barnett et al. [119]. Fluorescence, size-exclusion chromatography, mass spectrometry, near-UV circular dichroism, and Raman spectroscopy were used to understand selective oxidation in six mAbs. They found Trp oxidation affects mAb higher-order structure, and that the “toolbox” approach provided not only a comprehensive understanding but also multiple markers to monitor Trp oxidation.

Post-translational modifications, such as glycation or amino acid oxidation, are important contributors to therapeutic protein structure, function, toxicity, and stability. Detection and quantification of glycation is another emerging application of Raman spectroscopy, for understanding its role in diabetes as well as providing a quality control measurement in therapeutic protein production. Two papers by McAvan et al. [120, 121] demonstrate the feasibility of Raman microscopy to measure protein glycation. The first paper incubated lysozyme and albumin, serving as model proteins, in glucose solution for 30 days [121]. Raman and Fourier-transform infrared (FTIR) were used to understand the potential of lysozyme’s six lysine sites and N-terminus for glycation. FTIR and Raman data were compared to mass spectrometry and a partial least squares regression model was developed over a range of 0–100% glycated samples from 19 datasets. Principal components analysis was used to look at correlations within the Raman data. The results indicate that the experimental protocol could be used to assess glycation, with possible Raman glycation markers at ~ 1040 cm^−1^ and 1121 cm^−1^. The latter paper demonstrated Raman’s ability to measure post-translation modifications, aggregation, and fragmentation in forced degradation samples of IgG4 molecules [120]. Finally, Raman spectroscopy can be used to rapidly optimize protein crystallization conditions, and confirm the molecular structure and purity of a crystallized protein product [89, 122]. Raman microscopy of lysozyme under native and denaturing conditions revealed structural changes from the monomer and oligomeric (fibril) states [123]. The amide I envelope ~ 1655 cm^−1^ and skeletal band ~ 930 cm^−1^ showed α-helix structure in the oligomer state similar to the monomer, but broadening of the band ~ 505 cm^−1^ from disulfide bonds indicated loss of tertiary structure. These data enabled the authors to propose a mechanism of lysozyme fibril formation that involved a “zipping” of local hydrophobic residues to stabilize the fibril. Collectively, these Raman and UV-resonance Raman studies show the breadth of possible applications for integrating Raman spectroscopy into higher-order structure, formulations, and therapeutic protein composition studies.

## Conclusions and perspective

Raman is an essential analytical technology with proven benefits from basic science understanding in the laboratory and supporting agile process development to ensuring consistent process and product quality during commercial manufacturing. Emerging upstream and downstream biopharmaceutical applications demonstrate the utility of Raman throughout a biopharmaceutical’s lifecycle. In upstream applications, new reports extend Raman’s utility into monitoring process parameters such as amino acids, pH, and conductivity in addition to frequently reported glucose and lactate. Application success and rapid return on investment are realized in Raman-based feedback control of glucose feeding, which enables in-process corrections, allows process intensification, and ensures process and product quality. We also see continued work on model scalability for a cell line and transferability across different cell lines and process conditions. While most work focuses on suspension cultures, we anticipate more studies in microcarriers and adherent cell cultures. The established history of Raman spectroscopy in protein analysis, formulation support, and biomedical research form the scientific basis for downstream processing and biopharmaceutical formulation applications in an industrial setting. We anticipate new reports in downstream, technology transfer, and biopharmaceutical product quality in the upcoming years. We are also enthusiastic about process Raman applications in continuous biomanufacturing, cell and gene therapy, automated Raman monitoring of high-throughput miniaturized bioprocesses, increased automation, and further integration of Raman into process control applications.
